# Myogenic Potential of Whole Bone Marrow Mesenchymal Stem Cells *In Vitro* and *In Vivo* for Usage in Urinary Incontinence

**DOI:** 10.1371/journal.pone.0045538

**Published:** 2012-09-21

**Authors:** Monica Gunetti, Simone Tomasi, Alessandro Giammò, Marina Boido, Deborah Rustichelli, Katia Mareschi, Edoardo Errichiello, Maurizio Parola, Ivana Ferrero, Franca Fagioli, Alessandro Vercelli, Roberto Carone

**Affiliations:** 1 Paediatric Onco-Haematology, Stem Cell Transplantation and Cellular Therapy Division, Regina Margherita Children’s Hospital, Turin, Italy; 2 Department of Anatomy, Pharmacology and Forensic Medicine, University of Turin, Turin, Italy; 3 Neuroscience Institute Cavalieri Ottolenghi, Orbassano, Turin, Italy; 4 Department of Neuro-Urology, CTO - Maria Adelaide Hospital, Turin, Italy; 5 Department of Paediatrics, University of Turin, Turin, Italy; 6 Department of Experimental Medicine and Oncology – Interuniversity Center for Hepatic Pathophysiology, University of Turin, Turin, Italy; Universidade de Sao Paulo, Brazil

## Abstract

Urinary incontinence, defined as the complaint of any involuntary loss of urine, is a pathological condition, which affects 30% females and 15% males over 60, often following a progressive decrease of rhabdosphincter cells due to increasing age or secondary to damage to the pelvic floor musculature, connective tissue and/or nerves. Recently, stem cell therapy has been proposed as a source for cell replacement and for trophic support to the sphincter. To develop new therapeutic strategies for urinary incontinence, we studied the interaction between mesenchymal stem cells (MSCs) and muscle cells *in vitro*; thereafter, aiming at a clinical usage, we analyzed the supporting role of MSCs for muscle cells *in vitro* and in *in vivo* xenotransplantation. MSCs can express markers of the myogenic cell lineages and give rise, under specific cell culture conditions, to myotube-like structures. Nevertheless, we failed to obtain mixed myotubes both *in vitro* and *in vivo*. For *in vivo* transplantation, we tested a new protocol to collect human MSCs from whole bone marrow, to get larger numbers of cells. MSCs, when transplanted into the pelvic muscles close to the external urethral sphincter, survived for a long time in absence of immunosuppression, and migrated into the muscle among fibers, and towards neuromuscular endplates. Moreover, they showed low levels of cycling cells, and did not infiltrate blood vessels. We never observed formation of cell masses suggestive of tumorigenesis. Those which remained close to the injection site showed an immature phenotype, whereas those in the muscle had more elongated morphologies. Therefore, MSCs are safe and can be easily transplanted without risk of side effects in the pelvic muscles. Further studies are needed to elucidate their integration into muscle fibers, and to promote their muscular transdifferentiation either before or after transplantation.

## Introduction

Urinary incontinence (UI), defined as the complaint of any involuntary loss of urine, represents an increasingly frequent pathological condition, which occurs in 30% females and 15% males aged over 60. Stress urinary incontinence (SUI) is “complaint of loss of urine on effort or physical exertion or on sneezing or coughing” while urodynamic stress incontinence (USI) is “involuntary leakage during filling cystometry, associated with increased intra-abdominal pressure, in the absence of a detrusor contraction” [1]. An important cause of SUI is a progressive decrease of rhabdosphincter cells due to increasing age, which is caused by physiological apoptosis [2]. The significant progressive decrease in the number of striated muscle cells in the rabdosphincter with ageing represents a pathogenetic hypothesis for the high incidence of incontinence. Several different etiologies have been identified, such as ageing, obesity, multiple labors and prostatic surgery. The pathogenetic mechanism consists in a damage to the pelvic floor musculature, connective tissue and/or nerves. The outcome is urethral hypermobility and sphincter deficiency, isolated or in combination. Pharmacology failed to treat the disease: for instance, alpha agonists had no significant effect on the incontinence [3]. Several bulking agents have been proposed [3], [4]. Even though some of them have improved continence in selected patients, many unwanted side effects have been described [4], [5]. Moreover, treatment often fails in the long-term [6]. Non-invasive treatment is likely to be offered in mild cases and may entail pelvic floor muscle re-education.

Surgical approaches such as sling procedures and bladder neck suspensions are more efficacious [7]. These procedures are effective but can bear side effects such as bladder and urethral lesions or urinary retention. Other options, such as adjustable continence therapy (pro-ACT, consisting of two balloons placed at the bladder neck) may be considered in specific situations [8]. The optimal surgery has not yet been clearly established and current therapies for SUI do not treat pathophysiologic causes [9]. Future treatment paradigms may prioritize improving urethral sphincter function rather than urethral support [10].

Recently, stem cell therapy emerged as a revolutionary and useful tool for many diseases [11]. Therefore, it has been proposed as a source for cell replacement in SUI and for trophic support to the sphincter [4], [12]. Some studies pointed out the possibility of treating SUI with stem cells from muscle biopsies [13]. Mesenchymal Stem Cells (MSCs) represent an alternative source for cell therapy. MSCs can be isolated from different organs or tissue compartments including bone marrow (BM), umbilical cord blood, umbilical cord stroma (Wharton’s jelly), placenta, adipose tissue, amniotic fluid, dental pulp and many others [14].

In an autologous context of regenerative medicine bone marrow-derived mesenchymal stem cells (BM-MSCs) are ideal for transplantation: they are easily collected from the same patient and are renewable, multipotent cells. They can differentiate into various mesodermic cytotypes, including osteoblasts, chondrocytes, adipocytes [15], [16], tenocytes, myocytes and stromal cells, and act as minipumps, delivering trophic factors and immunomodulatory molecules [17]. Moreover, they present reduced risk of eliciting immunoreaction, due to their immunomodulatory potential, and minor side effects in terms of tumorigenesis than embryonic stem cells. The particular characteristics and high plasticity of BM-MSCs make them ideal candidates in cell therapy strategies to treat a number of degenerative [18], [19] and post-traumatic diseases caused by damage or cell loss [20], [21].

In an attempt to develop new therapeutic strategies for urinary incontinence we studied the interaction between MSCs and muscle cells *in vitro*; thereafter, aiming at a clinical usage in SUI, we analysed the supporting role of MSCs for muscle cells *in vitro* and in *in vivo* xenotransplantation. In keeping with the aim of treating SUI with MSCs, we tested a system to collect BM-MSCs from whole bone marrow, in order to get larger numbers of cells. We aimed a) to study the potential for myogenic differentiation *in vitro* of BM-MSCs alone and in co-culture with muscle cells, and b) to study their survival, proliferation and differentiation *in vivo* following transplantation into the perineal muscles. First of all, we isolated, cultured and characterized mouse BM-MSCs to be cocultured with C2C12 mouse skeletal myoblasts [22]. Then, we characterized the myogenic potential of human BM-MSCs *in vitro*, to be transplanted into the pelvic muscles of male rats.

## Materials and Methods

### a) Co-culture of Mouse BM-MSCs and Myoblasts

#### Isolation and culture of mouse BM-MSCs (BM-mMSCs)

BM-mMSCs were obtained from BCF1 mice, expressing enhanced green fluorescent protein (EGFP) under the beta-actin promoter, kindly provided by Dr. M. Okabe (Osaka University, Suita, Japan [23]). Seven-to-nine week-old EGFP mice were killed by cervical dislocation; their tibias and femurs were cleared of muscle and connective tissue. Bone marrow cells were aspirated using a 22-gauge needle, and washed twice for 5 minutes each by centrifugation at 150 g in Eagle’s alpha minimum essential medium (a-MEM; Sigma, St. Louis, MO, USA), containing 2 mM L-glutamine (Invitrogen-Gibco¸ Carlsbad, CA, USA), 100 U/ml penicillin and 100 µg/ml streptomycin (Invitrogen-Gibco). Cells were seeded in polystyrene 19.5 cm^2^ dishes (BD Biosciences) pre-treated with a coating of foetal bovine serum (FBS; Sigma); cells were grown in a-MEM supplemented with 10% FBS, in a humidified atmosphere of 95% air with 5% CO_2_ at 37°C. Medium was replaced on day 4 to remove free floating cells, and then replenished every 2–3 days. At 10 days *in vitro*, adherent cells were retrieved by trypsinization (Trypsin, Invitrogen-Gibco) and immunodepleted of CD11b-positive granulocytic cells by magnetic cell sorting: cells were incubated with MicroBeads conjugated to monoclonal rat anti-mouse/human CD11b antibody (Miltenyi Biotec GmbH, Bergisch Gladbach, Germany) and loaded onto a MACS column (Miltenyi Biotec). CD11b-negative cells were harvested, washed and re-plated onto dishes as described above. When the cells were near confluence, they were washed with a-MEM medium and incubated with trypsin for 5 min at 37°C. Trypsin was neutralized by adding fresh complete medium. The cellular suspension was diluted 1∶2 at each passage.

#### C2C12 cell culture and differentiation

C2C12 mouse skeletal myoblasts from ATCC (ATCC-LGC Standards S.r.l., Sesto San Giovanni (MI), Italy) were cultured in Dulbecco’s Modified Eagle’s Medium (DMEM) high glucose (Sigma) with 10% FBS (Sigma), containing 2 mM L-glutamine, 100 U/ml penicillin and 100 µg/ml streptomycin (all purchased from Invitrogen-Gibco). Cells were maintained in a humidified atmosphere of 95% air with 5% CO_2_ at 37°C. When at confluence, cells were detached with trypsin, as previously described, and split 1∶5. In order to allow C2C12 myoblasts to differentiate into myotubes, the serum concentration was reduced to 3% and maintained in culture with media changes every 2 days.

#### Co-culture of C2C12 and BM-mMSCs

C2C12 cells and BM-mMSCs were co-cultured at the ratio of 1∶4, in DMEM-high glucose (Invitrogen) containing 10% FBS (Sigma), containing 2 mM L-glutamine, 100 U/ml penicillin and 100 µg/ml streptomycin (Invitrogen). The ratio of 1∶4 of C2C12/BM-mMSCs was chosen after a literature survey, considering the cellular growth respectively very fast for C2C12, and markedly slow for murine MSCs. Several different ratios have been proposed for the coculture of MSCs and myoblasts [24]–[27]. After 2 days of co-culture, growth medium was substituted with differentiation medium, consisting of DMEM high glucose (Sigma) with 3% FBS (Sigma). Fresh differentiation medium was added every 2–3 days until 4 weeks.

#### Immunofluorescence of cell cultures

Cells were fixed in 4% paraformaldehyde (PFA) in PBS for 15 min at room temperature. Cells were then permeabilized with PBS containing 0.3% Triton X-100 for 10 min. After blocking unspecific binding sites with 1% BSA in PBST for 30 min at room temperature, cells were incubated in the primary antibody (monoclonal anti-human desmin, made in mouse, 1∶50; Dako, Denmark) made up in the same solution at 4°C overnight. After washing in PBS, samples were incubated in 1∶200 cyanine 3-conjugated secondary antibodies anti-mouse (Jackson ImmunoResearch Laboratories, West Grove, PA, USA) in PBS, for 1 hour at room temperature. For counter staining, cells were incubated 2 min with 0.001 g/ml Bisbenzimide in PB 0.1 M and rinsed with PBS. Finally coverslips were mounted with a drop of PB 0.1 M. The samples were examined with a Leica TCS SP5 confocal laser scanning microscope (Leica Microsystems, Milan, Italy).

### b) Characterization of Myogenic Potential of BM Human MSCs (BM-hMSCs)

#### BM-hMSC isolation and expansion

Whole Bone Marrow (wBM) hMSCs were isolated from BM obtained by aspiration from the posterior iliac crest of five healthy donors after written informed consent in accordance with the approval of the ethics committee of the hospitals OIRM-S.Anna-Mauritian order. BM-hMSC frequency in BM was about 1/10^4^ cells [28]. Briefly, wBM was seeded at a density of 100,000/cm^2^ in Mesenchymal Stem Cell Growth Medium (MSCGM) medium (Lonza, Basel, Switzerland) in 75 or 150 cm^2^ T-flasks and maintained at 37°C with an atmosphere of 5% CO_2_. After 5 days, the non-adherent cells were removed and re-fed every 3–4 days; at confluence, they were detached, and re-plated at 1,000 cells/cm^2^ density for one to four passages [29]. The cells were analysed for immunophenotype, differentiation potential and were used for all *in vivo* and *in vitro* experiments. The cellular expansion growth rate of MSCs was evaluated by cell count in a Burker Chamber at each passage and expressed in terms of population doubling (PD) using the formula log N/log 2, where N is the cell number of the confluent monolayer divided by the initial number of cells seeded [28].

#### Cytofluorimetric analysis

The characterization of wBM-hMSCs was performed by flow cytometry analysis at each passage. Briefly, 200,000–250,000 cells were stained for 20 minutes with anti-CD45 Fluorescein Isothiocyanate (FITC), CD14 Phycoerythrin (PE), CD34 Allophycocyanin (APC), CD90 FITC, CD73 PE, CD29 APC, CD44 FITC (Becton Dickinson, San Jose, CA, USA), CD105 FITC (Immunostep, Salamanca, Spain). Viability was evaluated by adding 7- aminoactinomycin D (AAD) (Becton Dickinson). The labeled cells were thoroughly washed with PBS and analyzed on Facs Canto II (Becton Dickinson) with the Facs Diva software program. The positive cell percentage was calculated using cells stained with Ig FITC/PE/APC as a Negative Control.

#### Differentiation potential assay (data not shown)

For differentiation experiments, wBM-hMSCs were cultured in osteogenic, adipogenic and chondrogenic medium (Lonza) according to the manufacturer’s instructions. Briefly, 20,000 and 50,000 cells were plated in a T-25 flask for osteogenesis and adipogenic culture conditions respectively, allowing the cells to adhere to the culture dish for 24 hours in MSC medium (Lonza). To induce osteogenesis and adipogenesis, the medium was replaced with specific complete induction medium (Lonza). After 21 days, osteogenic differentiation was demonstrated by the accumulation of calcium (crystalline hydroxapatite detection by Von Kossa staining) in separated cells plated in chamber slides in the same culture conditions.

For the adipogenic differentiation, adipogenic induction and maintenance medium were alternatively used every 3–4 days and the presence of intracellular lipid vesicles visible after 2–3 weeks’ culture was assessed by Oil Red O staining.

For chondrogenic differentiation, an aliquot of 250,000 cells was washed twice in incomplete chondrogenic medium (Lonza) in 15 ml polypropylene culture tubes. Finally, the cells were resuspended in complete chondrogenic medium, centrifuged and, without aspirating the surnatant, the tubes were incubated at 37°C, in a humidified atmosphere of 5% CO_2_. Chondrogenic differentiation was obtained growing cells as free-floating aggregates in suspension culture with Transforming Growth Factor (TGF)-beta3. The pellet was paraffin-embedded and stained with Alcian Blue to identify the presence of hyaluronic acid and sialomucin.

#### Myogenic differentiation

To test the differentiation potential of wBM-hMSCs towards myogenic lineage we induced myogenic differentiation of BM-hMSCs at the first–fourth passages as follows. We tried to translate, to wBM-hMSCs, the promising differentiating conditions used by Gowronska-Kozak B and coll., in a mouse model [30]. To this purpose we used all materials and reagents suitable for human cell growth. Briefly, 5,000 wBM-hMSCs/well were seeded on collagen, laminin, gelatin and fibronectin coated matrix (Becton Dickinson Labware, NJ, USA) in Dulbecco’s Modified Eagle Medium (DMEM–F12, Invitrogen, San Giuliano Milanese, Milan, Italy) supplemented with 15% of FBS. Forty-eight hours after plating, the medium was removed and the cells were washed with Hanks’ Balanced Salt Solution (HBSS; Sigma, Saint Louis, MI, USA). The cells were then cultured in DMEM-F12 with 0.1% of Insulin Transferrin Selenium (ITS; Invitrogen), 5% of FBS and 0.2 mg/ml Epidermal Growth Factor (EGF; Sigma) for 7 days.

#### Immunocytochemistry

Since we obtained the best differentiation towards myogenic lineage by culturing wBM-hMSCs on laminin coated matrix, using DMEM F12 with ITS, EGF and 5% FBS, we evaluated myogenic differentiation of basal wBM hMSCs and laminin cells. We performed immunocytochemistry analysis for myogenic markers at 7 days *in vitro*, when we observed the presence of binucleated cells. Human Skeletal Muscle Myoblasts (HSMM, Lonza) were used as positive control. HSMM were cultured in Skeletal Muscle Myoblast Cell Medium-2 (Lonza) according to data sheet.

The cells were fixed and permeabilized with acetone-methanol (1∶1) at −20°C for 20 minutes. Non-specific binding sites were blocked with 0.1% human serum albumin (HSA) in PBS 1X. The cells were incubated with the primary antibody: anti-Myogenin (1∶500, Chemicon, Temecula, USA), anti-Desmin (1∶20, Chemicon); anti-Sarcomeric Actin (SA) (1∶100, Dako Cytomation, Glostrup, Denmark); anti-Alpha Smooth Muscle Actin (α-SMA) (1∶100, Dako); anti-Myosin (1∶10, Sigma, Taufkirchen Germany). Binding was revealed by CY3-coupled anti-rabbit (1∶1000, Immunological Sciences, Rome, Italy) and Alexa fluor 488-coupled anti-mouse (1∶200, Molecular Probes, Oregon, USA) secondary antibodies. All incubations were performed for 1 hour at room temperature or at 4°C, overnight. Before each step, the cells were washed in 1% HSA PBS.

#### PCR for L-type Ca2+ ion channel

We performed Ca^2^+ ion channel analysis in wBM-hMSCs compared to cells differentiated on laminin-coated matrix. Reverse Transcriptase-Polymerase Chain Reaction (RT-PCR) for *calcium ion channel (α1C, α1D and α1S)* expression was performed in 25 µl reaction mixture as follows: 100 ng of cDNA, 1X reaction buffer, 1.25 mM MgCl2, 0.2 mM of each deoxynucleotide triphosphate, 0.6 µM of each forward and reverse primer, and 1.25 U of Taq Gold Polymerase. The forward and reverse primers were previously reported [31]. The amplification conditions were: 94°C for 10 min, 40 cycles of 94°C for 45 s, annealing for 1 min, 72°C for 1 min and a final extension at 72°C for 7 min. The annealing temperatures were 56°C for α1C subunit, 58°C for α1D and 60°C for α1S. The PCR products were electrophoresed through a 1.5% agarose gel and amplicon bands were visualized by ethidium bromide staining using Gel Doc 2000 (Biorad, UK).

### c) Transplantation of BM-hMSCs in Rats

#### Ethical statement

Male adult Sprague-Dawley (Harlan, Italy) weighing 250–350 g were used in this study. All animal experimental procedures were approved and carried out in strict accordance to European Community Council Directive 86/609/EEC (November 24, 1986), Italian Ministry of Health and University of Turin institutional guidelines on animal welfare (law 116/92 on Care and Protection of living animals undergoing experimental or other scientific procedures; authorization number 17/2010-B, June 30, 2010) and ad hoc Ethical Committee of the University of Turin.

#### Prelabelling of BM-hMSCs

Cells expanded for the first 3–8 passages were labelled by adding to the medium 10 µg/ml bisbenzimide (Sigma, St. Louis, MO, USA), which binds DNA, 24 h before transplantation. Then the cells were detached with Trypsin/EDTA, washed and re-suspended in saline solution to obtain a final concentration of 50,000 cells/µl to be used for transplantation.

#### Study design and surgical procedures

All experimental procedures on live rats were done according to the European Communities Council Directive (86/609/EEC) and following the guidelines for care and use of laboratory animals as published by Italian Ministry of Health (DDL 116/92). Animals had free access to food and water. All efforts were made in order to minimize the number of animals used and their suffering. Rectal temperature was maintained at 36.5–37.5°C throughout the procedure with a heating pad. Sprague-Dawley rats were used to assess survival, migration and differentiation of BM-hMSC after transplantation into striated muscles. Rats were randomly divided into 3 groups, which were evaluated 24 hours (n = 3, group A), 1 month (n = 5, group B) or 4 months (n = 4, group C) after stem cell transplantation. Since it was impossible to limit the spread of stem cells from the external urinary sphincter (EUS) in rats, due to its extreme thinness, we decided to inject stem cells into the whole group of perineal muscles, including the ischiocavernosus and the bulbocavernosus muscles. Briefly, rats were anaesthetised with 2% isoflurane vaporised in a 70/30 mixture of N_2_O/O_2_ and delivered using a face mask. Under the operating microscope (Carl Zeiss Inc., Jena, Germany) the scrotum was incised on the midline and the testicles carefully displaced laterally. After dissecting surrounding tissue and fascia, the bulbar urethra was identified and the perineal muscles were visualized. Using a 10-µl Hamilton syringe (27.5 G), two injections (2 µl of a suspension of 50,000 cells/µl each) per rat were performed under microscopic guidance into perineal muscles close to the urethra. The optimal concentration of MSCs in order to avoid cell damage during injection and to allow the flow of the solution through the needle had been determined in previous studies [19]. After stem cell administration, surgical wounds were sutured and all rats were returned to the cages. The time required for the procedure was approximately 15 minutes.

#### Tissue preparation

Animals were killed with an overdose of anaesthetic at different time points after transplantation (24 hours to 4 months) and perfused through the ascending aorta with saline followed by the fixative (PFA 4% in 0.1 M phosphate buffer, pH 7.4). Bulbar urethra with the surrounding perineal muscles was collected from each rat, post-fixed in the same fixative for one day and cryoprotected in 30% sucrose in PBS overnight. Samples were then sectioned on the cryostat in 18 µm-thick serial sections, mounted on chrome alum-coated slides, stained with hematoxylin eosine to recognize transplantation site along with anatomic reference points such as urethral wall, external urinary sphincter, observed and photographed at the light microscope.

#### Immunofluorescence and histochemistry

Sections were rinsed in PBS, then permeabilized with PBS containing 0.3% Triton X-100, and successively washed three times in PBS. Non specific binding sites were blocked by incubating sections 45 minutes at room temperature in 10% normal donkey serum (Sigma-Aldrich) in 0.3% PBS-Triton X-100. Slices were immunoreacted overnight at +4°C in monoclonal mouse anti-human desmin (1∶50, Dako, Denmark) or rabbit polyclonal anti-Ki67 (1∶400, Novocastra, UK). Desmin immunoreactivity was considered as an early marker of muscle differentiation [32]. After washing in PBS, sections were incubated three hours at room temperature with cyanine-2-conjugated or cyanine-3-conjugated anti-rabbit secondary antibodies. Alfa-bungarotoxin (α-BTX) histochemistry was performed to detect acetylcholine receptors (Ach-Rs) in skeletal muscles sections as follows: sections were rinsed in PBS then incubated 3 hours in a PBS solution containing Alexa Fluor 555-conjugated α-bungarotoxin (1∶1000, Molecular Probes, Invitrogen, USA), then washed in PBS and mounted on 2% gelatin-coated slides. All processed sections were then observed with a Leica TCS SP5 laser scanning confocal microscope (Leica S.p.A., Milan, Italy).

#### Semi-quantitative analysis

In order to evaluate stem cell survival and density within the site of transplantation, sections were visualized at 10–20× magnifications under a Nikon Eclipse 800 epifluorescence microscope (Nikon S.p.A., Sesto Fiorentino, FI, Italy) and images were acquired and digitalized using a Nikon Coolpix E995 digital camera. Images were then processed with NIH ImageJ (software version 1.5.0, freely available online at http://rsbweb.nih.gov/ij). The site of transplantation was manually outlined at the computer-interfaced microscope at the 10× lens, thus obtaining a measure of area and perimeter. The motorized stage (Märzhäuser, Wetzlar, D) of the microscope allowed a 0.1 µm accuracy on the xy coordinates. At 24 hours all the stem cells were concentrated in the area of injection. Afterwards, we used the area of the injection site as an indirect estimate of cell scattering along the muscular tissue for any considered time point, expressed as the largest value measured through the sections for each sample for comparisons. Results are presented as percentage ± SEM (labelled cells/total number of grafted cells).

## Results

### Co-culture Experiments

**Figure 1:**
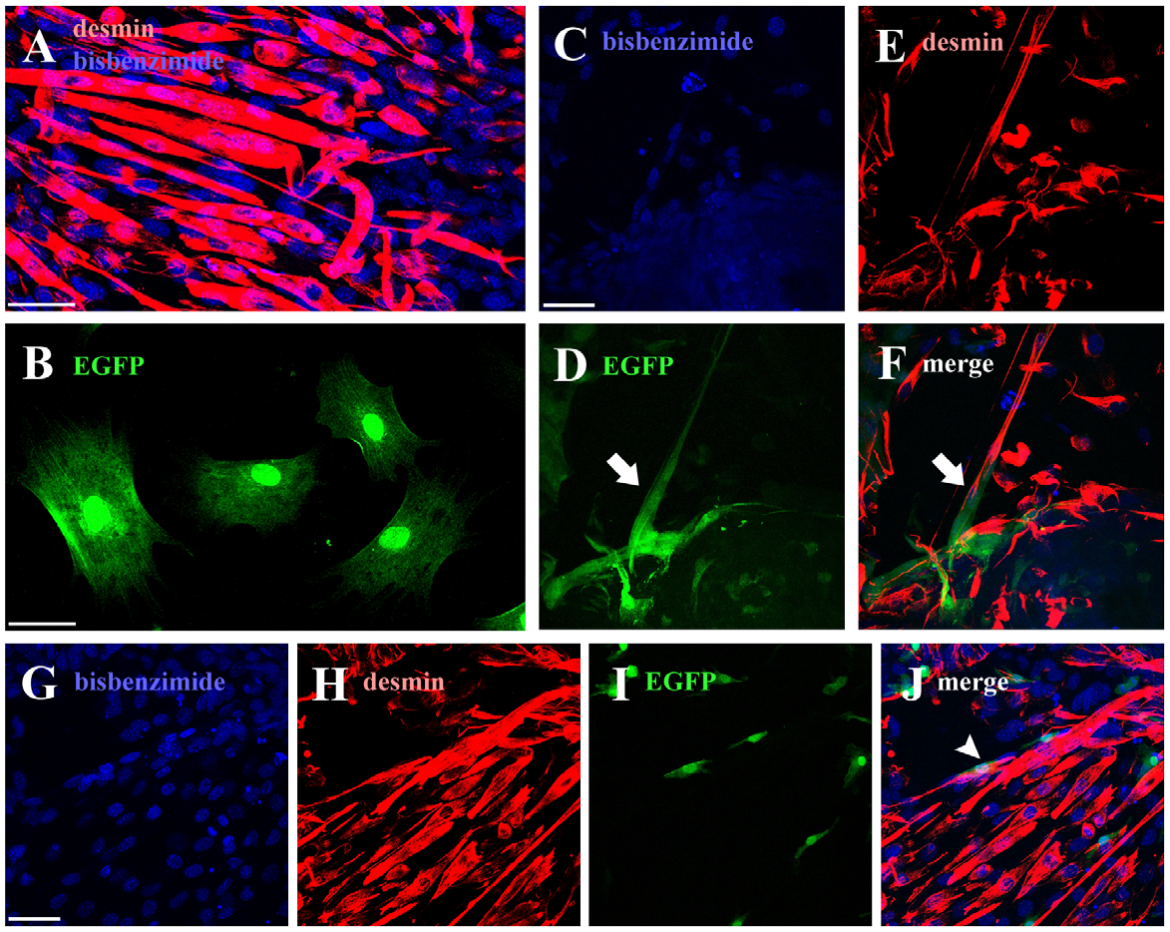
Mouse BM-mMSCs. (A) Desmin-positive multinucleated myotubes (nuclei labelled in blue with bisbenzimide) derived from the fusion of C2C12 myoblasts. (B) EGFP-MSCs, plated alone, display a fibroblast-like shape. (C–F) In co-culture with C2C12 cells (desmin-positive, labelled in red), MSCs (green) show long cytoplasmatic processes (arrow). (G–J) EGFP-MSCs adhere to desmin-positive myotubes (arrowhead). Scale bar = 50 µm.

In order to evaluate the interplay between BM-MSCs and muscle cells *in vitro*, we co-cultured mouse BM-mMSCs and C2C12 cells. After one week in differentiation medium, myotubes derived from the fusion of C2C12 myoblasts could be observed, showing the typical elongated multinucleated structure (Fig. 1A), occasionally presenting contractions. In addition, all C2C12 cells resulted desmin-positive (Fig. 1A), the intermediate filament particularly localized to the Z-band in sarcomeres. EGFP-positive BM-mMSCs alone displayed a fibroblast-like morphology (Fig. 1B): on the contrary, in co-culture with C2C12 cells, BM-mMSCs frequently appeared elongated, with long cytoplasmic processes (Fig. 1C–F). BM-mMSCs generally lined up following the fibers orientation and adhering to the myotube membranes (Fig. 1G–J). Nevertheless, fusion between BM-mMSCs and C2C12 could not be observed *in vitro*; in addition, BM-mMSCs were desmin-negative, suggesting their tendency to remain undifferentiated. Taken together, these observations indicate that BM-mMSCs rather support muscle cells, than being involved into myotube formation.

### Characterization of wBM-hMSCs

**Figure 2:**
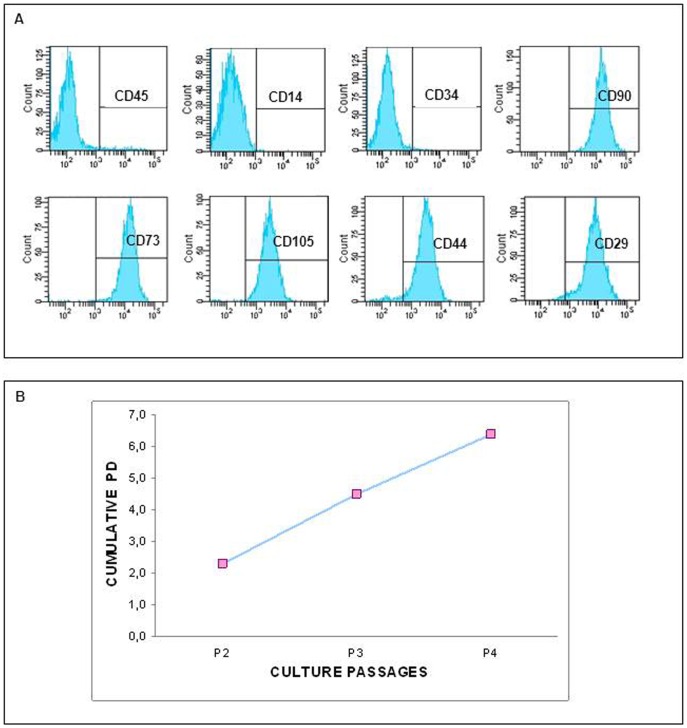
Cytofluorimetric and morfological analysis of a representative wBM-hMSCs. (A) Immunophenotypic analysis of wBM hMSCs showing the negativity of haematopoietic markers CD45, CD14, CD34, and the positivity of CD90, CD29, CD73, CD105, CD44. (B) Analyses of growth rate of wBM hMSCs in terms of cumulative PD. The graphic refers to the median cumulative values (1^st^ passage: median 2.3 - range 1.4–2.6; 2^nd^: median 4.5 - range 2.9–5.9; 3^rd^: median 6.4 - range 4.9–8.5).

**Figure 3:**
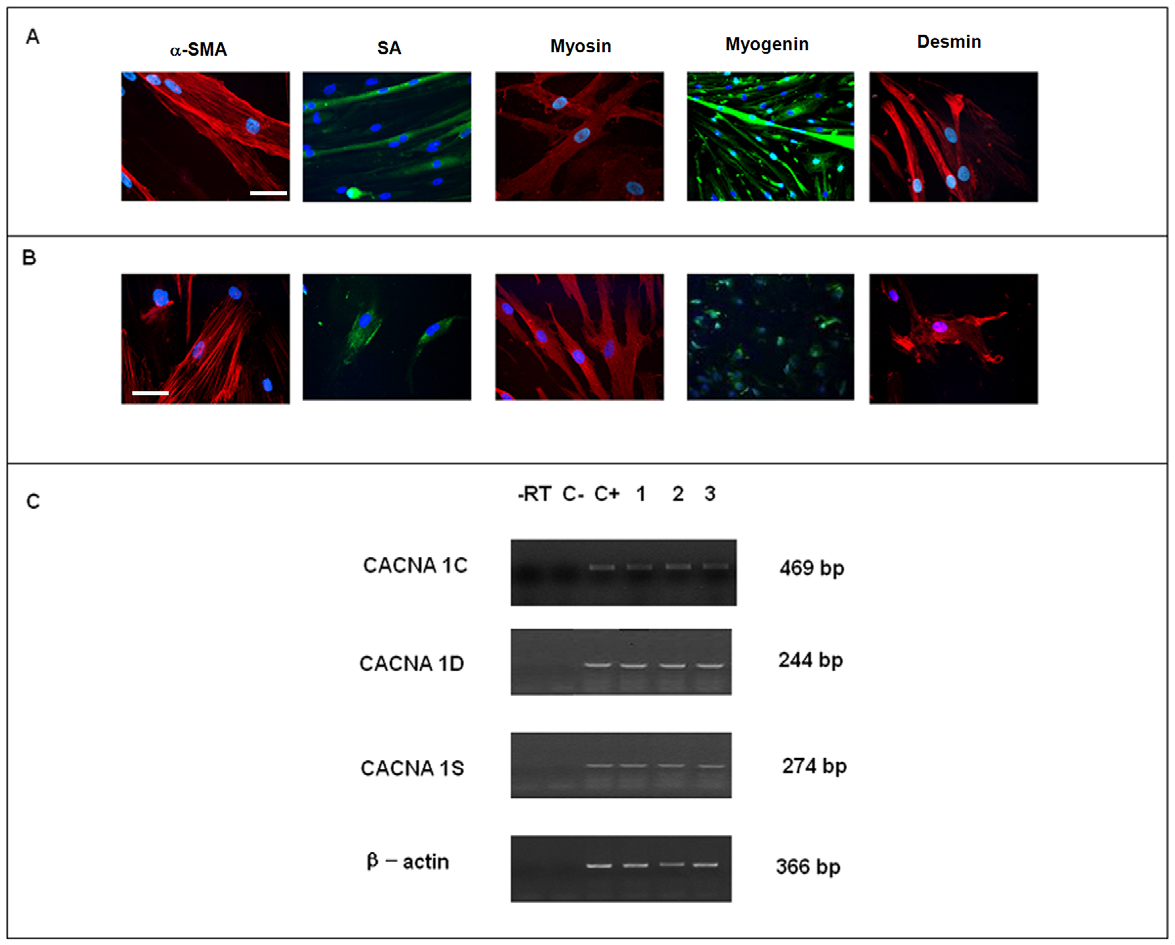
Immunofluorescence and Molecular analysis of *Calcium ion channel subunits* in wBM-hMSC. (A) Positive control normal Human Skeletal Muscle Myoblast (HSMM). Scale bar = 10 µm (alpha SMA) and 25 µm (SA, Myosin, Myogenin and Desmin). (B) Undifferentiated wBM hMSC analyzed at 28 days. Scale bar = 10 µm (a-SMA), 25 µm (SA, Myosin, and Desmin), 50 (Myogenin). (C) Original gels demonstrating amplification of calcium ion channel subunit transcripts in wBM hMSCs: -RT: control of reverse transcription without RT enzyme; C−: negative control (water); C+: positive control (HSMM); line 1: wBM hMSCs from first passage; line 2: wBM hMSCs from second passage; line 3: wBM hMSCs from fourth passage; β-actin, housekeeping gene.

With the view of treating SUI with MSCs, we tested a system to collect BM-MSCs from whole bone marrow, to get larger numbers of cells. wBM-hMSCs were characterized following guidelines of The International Society for Cellular Therapy [33]: as revealed by cytofluorimetric analysis, wBM-hMSCs did not express CD45, CD14, CD34 haematopoietic markers, whilst expressed high levels of CD90, CD105, CD73, CD29, CD44 (Fig. 2A) and had a large expansion of cells expressed in terms of cumulative population doubling (PD) during passages of culture (Fig. 2B). Moreover, wBM-hMSCs differentiated into osteoblasts, chondroblasts and adipocytes under specific differentiation media (data not shown). In order to use wBM-hMSCs as Cell Therapy Products (CTP) for SUI treatment we evaluated the expression of myogenic markers such as α-SMA, SA, Myosin, Myogenin, and Desmin. Similarly to human myoblast culture (HSSM), that can give rise to myotubes (Fig. 3A), wBM-hMSCs displayed positivity for the above mentioned myogenic markers (Fig. 3B).

### Differentiation of wBM-hMSC Toward Myogenic Lineage

**Figure 4:**
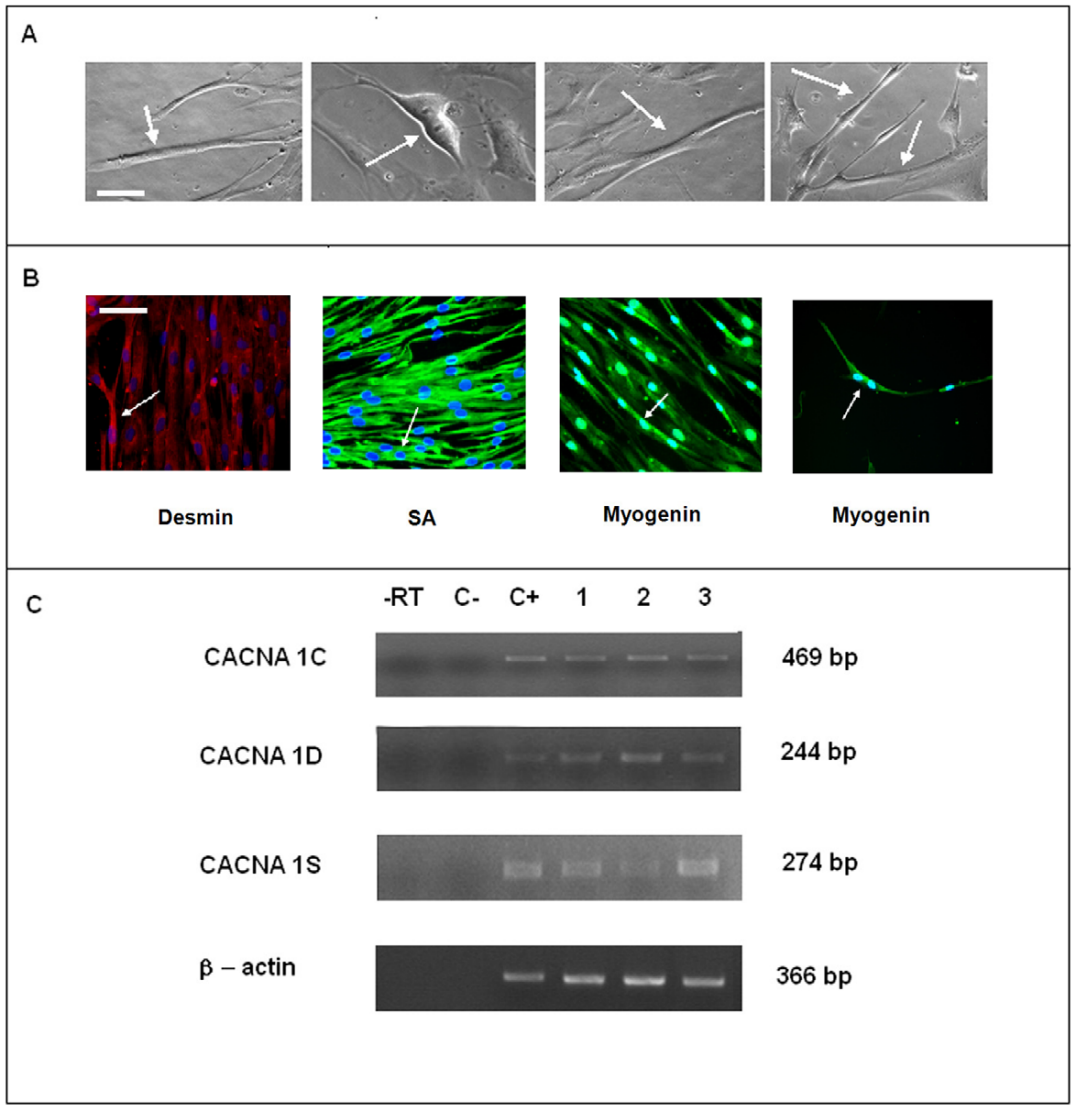
Myogenic differentiation on laminin matrix and *L-type Calcium ion channel subunits* analysis. (A) Phase contrast images of laminin cells: presence of some binucleated cells. Scale bar = 25 µm. (B) Immunofluorescence analysis confirmed the presence of a few binucleated structures positive for desmin, SA, myogenin in BM-hMSC cultured on laminin cells. Scale bar = 25 µm. (C) Original gels demonstrating amplification of *calcium ion channel subunit* transcripts in laminin cells: –RT: control of reverse transcription without RT enzyme; C−: negative control, water; C+: positive control, HSMM; line 1: control wBM-hMSCs; line 2: laminin cells cultured in DMEM-F12 supplemented with 15% FBS; line 3: laminin cells induced to myogenic differentiation with EGF for 7 days.

In order induce wBM-hMSCs to myotubes, we differentiated these cells using coating matrix as Gowronska-Kozak et coll reported in a mouse model [30]. To this purpose we used all materials and reagents suitable for human cell growth. Coated cells were monitored daily by phase contrast microscopy to evaluate myotube formation. During the first 48 hours, when the cells were maintained in DMEM F12 with 15% FBS, they showed a typical fibroblast-like phenotype. After medium replacement, we observed the presence of some binucleated cells at 7 days only after differentiation on laminin-coated matrix (Fig. 4A). Even though these differentiated cells continued to express the myogenic marker (Fig. 4B), we never observed myotube formation. Only a few binucleated structures (Fig. 4B) were positive for myogenic markers such as desmin, SA and myogenin.

### L-type Calcium Channel α-subunits Expression

On the basis of our immunohistochemical data, we investigated the expression pattern for L-Type calcium channel α-subunits (α1C, α1D and α1S), involved in myogenesis, in wBM-hMSCs and in cells differentiated on laminin-coated matrix. According with immunohistochemical results, above described, we found mRNA expression for all L-Type calcium channel α-subunits (α1C, α1D and α1S) in wBM-HMSC and in cells differentiated on laminin-coated matrix. In particular, high mRNA levels of L-Type calcium channel α1C, α1D and α1S subunits were detected at first, second and fourth culture passages in wBM-hMSCs (Fig. 3C), and also following culture on laminin cells in DMEM–F12 with 15% FBS, and in laminin cells induced to myogenic differentiation with ITS and EGF for 7 days (Fig. 4C). HSMM cells, human myoblasts that give rise to myotubes in culture, were used as positive control for the process of myogenesis involving L-Type calcium channel α-subunits (α1C, α1D and α1S) (data not shown).

### Survival and Migration of BM-hMSCs Transplanted into the Rat Pelvic Muscles

**Figure 5:**
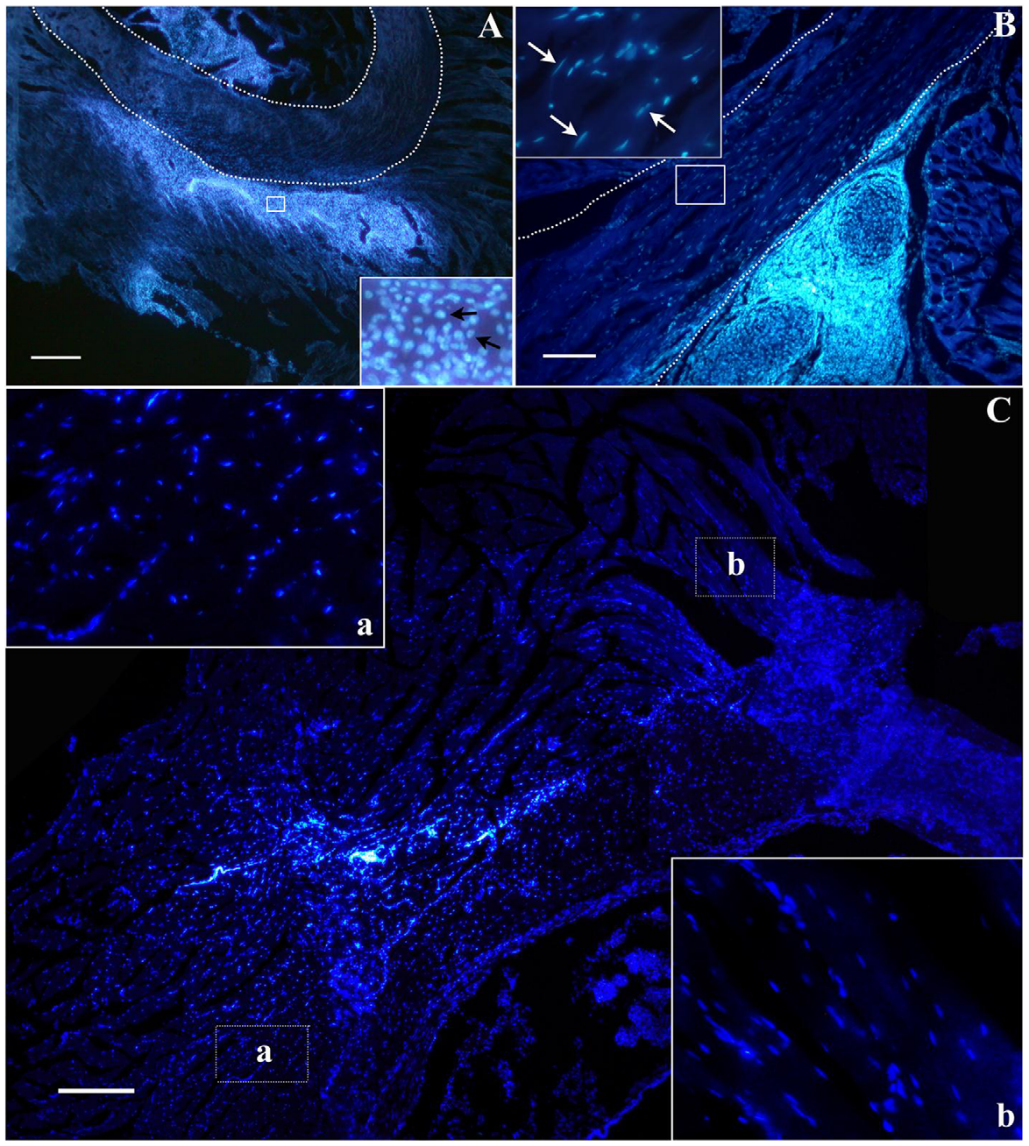
Bisbenzimide-stained BM-hMSCs (in blue) transplanted into rat bulbocavernosus muscle. (A) 24 hours after transplantation BM-hMSCs appear undifferentiated with a typical round shape (inset in A, scale bar = 500 µm); (B) one month after engraftment many BM-hMSCs with elongated shape are recognizable among muscular fibers (inset in B, scale bar = 100 µm). (C) At 4 months, migration toward muscle fibers is confirmed by elongated appearance of cells occupying peripheral position (inset b), whereas undifferentiated cells are observed in the core of graft (inset a). Scale bar = 500 µm.

We analyzed the outcome of stem cells transplantation at different time points in order to assess not only their survival within striated muscle, as evaluated at 24 hours, but also the morphological changes occurring over mid-term (1 month) or long-term (4 months). Bisbenzimide-stained BM-hMSCs could be observed at 24 hours within the bulbocavernosus muscle, at the boundary between muscle and urethral wall (Fig. 5A), extending across an area of approximately 1.8±0.3 mm^2^, with a typical round shape suggestive of an undifferentiated population of cells. One month after engraftment, BM-hMSCs were traced at injection site (across an area of 1.2±0.3 mm^2^) and, less numerous, at the external urinary sphincter, interspersed among muscular fibers, revealing a predominantly elongated shape (Fig. 5B), as also shown by adjusting the condenser lens to visualize the outline of the cells. At 4 months, BM-hMSCs spread over an area of 2.5±0.4 mm^2^, indicating an important migration toward muscle fibers (p<0.05). Injection site was still visible, although cellular density appeared lower than at shorter survival times; at this level, cells maintained a predominantly round shape, in contrast with the elongated appearance of cells at the periphery (Fig. 5C).

### Proliferation and Differentiation of BM-hMSCs Transplanted into the Rat Pelvic Muscles

**Figure 6:**
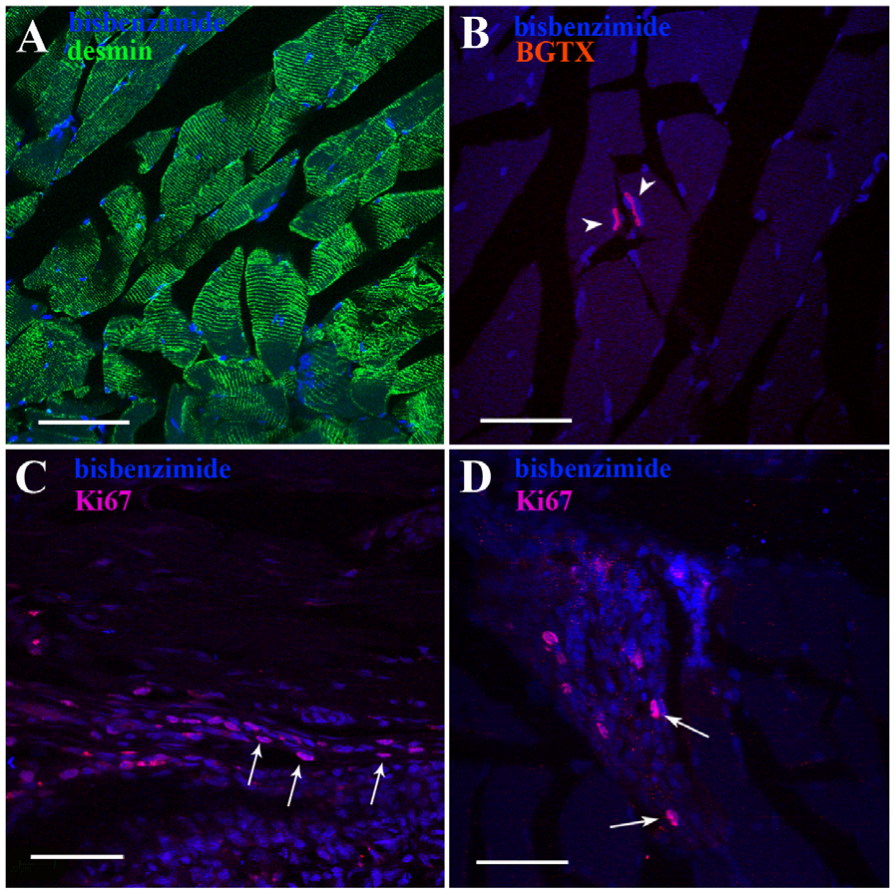
Integration of BM-hMSCs in the striate muscle. (A) Bisbenzimide-labelled BM-hMSCs (in blue) appear integrated into desmin-positive striated muscle fibers (in green). (B) At 4 months, several BM-hMSCs are located in close vicinity (arrowheads) to acetylcholine receptors (α-BTX staining, in red) (C). Proliferative profile of transplanted BM-hMSCs at one and (D) four months after transplantation, as revealed by Ki67 immunohistochemistry (in purple). Scale bar = 50 µm.

Muscle fibers were desmin-positive at any considered time point. Bulbocavernosus and ischiocavernosus muscles appeared as striated structures in which transplanted cells occupied a peripheral position, thus suggesting that BM-hMSCs can survive for long periods within muscular tissue, although no colocalization of markers was found, therefore we could not hypothesize any fusion between BM-hMSCs and striated muscle (Fig. 6A). α-BTX staining was diffuse one month after transplantation, whereas 4 months later we found that several BM-hMSCs were located close to Ach-Rs (Fig. 6B). Finally, we carefully assessed proliferation of BM-hMSCs with anti-Ki67 immunohistochemistry. One month after transplantation 13% ±2.1 of cells were Ki67-positive vs 9.8% ±3.5 (statistically not significant) counted 4 months later, thus indicating that in both cases low proliferation occurred and a tendency toward progressive loss of proliferative phenotype at 4 months could be assumed (Fig. 6C–D).

## Discussion

Rapid progress in biotechnology and medicine led to the development of new treatments and innovative medicinal products containing viable cells. CTPs are medicinal products for treating or preventing disease in human beings and their manufacturing process have to comply with the principles and guidelines of GMP for medicinal products for human use. Some studies suggested to treat SUI with stem cells from muscle biopsies [13]. MSCs may represent an alternative cell type for transplantation [4], since they may be easily collected in the same patient and, as shown in the present paper, expanded with minimal *ex-vivo* manipulation. Moreover, they can modulate the environment and support muscle fibers with trophic and immunomodulatory molecules.

The novelty of our approach consists in the usage of wBM MSC as CTP, in order to obtain a large amount of MSCs starting from few mL of BM aspirate, with a minimal *ex-vivo* manipulation [29]. Here, we performed *in vitro* studies to assess the capability of MSCs i) to differentiate into muscle cells or ii) to integrate with myogenic cell lines. To the first aim, we cultured wBM-hMSCs, the ones to be later used *in vivo*. To the second aim, we studied the mouse-to-mouse interaction, by culturing undifferentiated mouse MSCs with a myogenic cell line. In order to assess the feasibility of translation of MSC transplantation into clinics, and to exclude negative side effects, we performed an *in vivo* study in which we transplanted wBM-hMSCs in the rat perineal muscle. To our knowledge, this is the first study to transplant wBM-hMSCs, expanded without gradient separation, and to show the long term survival *in situ* of grafted cells in absence of immunosuppression and without side effects such as teratoma formation or uncontrolled cell proliferation.

Myogenic differentiation of MSCs has been previously reported, although some controversy remains [34]. Our results show that wBM-hMSC, even though they have a fibroblastoid, and not tubular, shape, express basally myogenic markers. Undifferentiated wBM-hMSCs express many myogenic markers, such as α-SMA, SA, myosin, myogenin, desmin, maintaining immunophenotypical characteristics and functions as multipotent cells. Our data are in agreement with those which show the expression of α-SMA, MYOD1 and MyHC in native MSC and AZA-exposed MSC, isolated by gradient density [35], and of desmin, myogenin, α-SMA by immunohistochemistry and RT-PCR in BM-hMSCs [36]. The expression of desmin by hMSCs seems to contradict with the failure of desmin expression by mMSC: to explain this finding, we hypothesize that human and murine MSCs can display a partially different cell-surface antigenic profile. For example, Stro-1, which is expressed by BM-hMSCs, has no known mouse counterpart [37]. Therefore, some myogenic markers might be more evident in human than in murine MSCs.

In addition to the *in vitro* expression of myogenic markers, we considered the expression of L-type channel α-subunits in BM-hMSCs to monitor their differentiation into muscle cells. Intracellular free Ca^2+^ is a fundamental biological signal regulating a number of cell functions. For instance, it plays an important role in controlling cell growth, transformation, secretion, smooth muscle contraction, sensory perception and neuronal signaling [38], [39]. Spontaneous intracellular free Ca^2+^ oscillations are present in MSCs. Nevertheless, their physiological functions in MSCs are still elusive [40], and Ca^2+^ channel expression in BM-hMSC remains not well elucidated. Kawano et al. [41] reported characteristic oscillations of membrane potential in hMSCs, isolated by gradient density, regulated by Ca^2+^ channel flux. Li et al. [42] described CACNA 1C mRNA expression in commercial hMSCs and Heubach et al. [43] reported a strong expression of the *L-type calcium channel* α1C subunit in all commercial hMSC samples but low or undetectable levels for other α1D and α1S subunits.

Since laminin cells were the only ones displaying a “myogenic like structure”, we evaluated the presence of L-type Ca^2+^ channel α-subunits (α1C, α1D and α1S) in these differentiated cells compared to wBM-hMSCs. All these subunits were expressed in wBM-hMSCs up to the fourth passage of culture at consistent levels between passages. Therefore, our data suggest their pivotal role in some prominent biological cell function. Relative to this, spontaneous Ca^2+^ oscillations created by inositol 1,4,5-triphosphate receptors (IP3Rs) have been recently observed in undifferentiated hMSCs during the G1 to S transition. Thus, these oscillations may play a role in the cell cycle progression and proliferation, possibly due to regulation of cyclin levels [44].

Additionally, we developed a valid differentiating protocol *in vitro*: in fact, under specific conditions (ITS, EGF and 5% FBS), we observed the presence of binucleated wBM-hMSCs and their positivity to several myogenic markers (α-SMA, SA, Myosin, Myogenin, Desmin).

Taken together, our data allow an overview of myogenic potential of wBM-hMSCs and could clarify some controversial basic concepts in the regenerative medicine field using BM-hMSCs. In fact, undifferentiated wBM-hMSCs, in addition to many early myogenic markers, express L-type calcium channel α-subunits and could *in vitro* differentiate into small myogenic like structures.

On the other hand, we show that the undifferentiated mouse MSCs do not form myotubes efficiently, and do not fuse with existing myotubes generated from co-cultured C2C12 cells. This is in apparent contrast with Beier et al. (2011) [27] who showed that MSC readily differentiate into myotubes *in vitro* and fuse at relatively high efficiency when co-cultured with native myotubes. Nevertheless, they used two kinds of differentiation media (DM): when cultured in DM alone, MSCs resulted positive to some myogenic markers, however “highest differentiation levels were observed in group G8, i.e. MSCs plus myoblasts cultured under stimulation with bFGF and dexamethasone”. On the contrary, no differentiation was reported when MSCs and the myogenic cell line L6 were cultured in bFGF/dexamethasone-free DM, their closest experimental condition to our. Our aim was to observe the interactions between MSCs and muscle cells in “standard conditions”, analyzing the integration and the supporting role of the former. Similarly, the human MSCs used for grafting were undifferentiated in order to observe their safety, their viability through time, and their capability of integrating into perineal muscles.

For the *in vivo* studies, we transplanted human MSC into the rat perineal muscles, as a preclinical study to establish clinical relevance and to exclude risks for teratoma formation or undesired cell proliferation, as already performed in transplantation studies into the CNS [19]. On the other hand, we did not observe in the past differences in the behavior of MSCs following mouse-to-mouse transplantation compared to xenogenic transplants in terms of cell proliferation or differentiation [20]. On the contrary, the *in vitro* studies were performed with GFP-positive mouse MSCs i) to exploit the interaction between MSCs and C2C12 mouse cell line in order to understand the potential for cell fusion and induction of MSC differentiation into muscle cells and ii) to allow the precise identification of MSCs for their green fluorescence.

When transplanted into the pelvic muscles close to the external urethral sphincter, BM-hMSCs survive for a long time in absence of immunosuppression, migrate into the muscle among muscle fibers, and towards neuromuscular endplates. Moreover, they show low levels of cycling cells, and do not infiltrate blood vessels. We never observed cell masses suggestive of tumorigenesis. Those which remain close to the injection site show an immature phenotype, whereas those in the muscle have more elongated morphologies. When in close proximity to muscle cells, MSCs display an elongated morphology aligned with muscle fiber orientation. This is in accordance with others, who showed that strings of peripheral MSC nuclei can be positioned along the length of preexisting fibers [45]. Therefore, BM-hMSCs are safe and can be easily transplanted without risk of side effects in the pelvic muscles. Further studies are needed to support their integration into muscle fibers, promoting their muscular transdifferentiation either before or after transplantation.

The long-term survival of BM-hMSCs in the rat pelvic muscles is not surprising. We have previously observed no immunoreaction after long-term grafting into the mouse spinal cord in an experimental model of amyotrophic lateral sclerosis [19]. This property of MSCs to prevent immunoreaction [46], and that of homing in the host bone marrow were also observed in Rhesus monkey-to-human transplantation [47].

Our data suggest that MSCs, even though they do not fuse with the host muscle cells, can migrate into the muscle and to the neuromuscular junction. MSCs can deliver immunomodulatory molecules and trophic factors [48] to support maintenance of the endplates and muscle/motoneuron trophism as already hypothesized by Canzi et al. [49] in a model of spontaneous motoneuron degeneration, the Wobbler mouse. These factors could be also useful in preventing age-related changes in the human urethral rhabdosphincter [50]. Promising results were obtained with MSCs collected from adipose tissue, recently transplanted periurethrally in two patients with stress urinary incontinence (SUI) after radical prostatectomy, reporting decreased incontinence [51]. On the other hand, transplantation of adipose tissue had no significant effects on females with SUI [52], contrarily to rats [53]. Kinebuchi et al. report that rat BM-MSCs, transplanted into the injured external urethral sphincter, differentiated into striated muscle cells and peripheral nerve cells, and improved functional outcome by reducing the abdominal leak point pressure in rats and rabbits [54]. A tissue engineered sling with BM-MSCs and seeded degradable silk scaffold can improve significantly SUI in female rats. Periurethral injection of adipose stem cells with controlled delivery of NGF improved significantly the functional outcome in an experimental model of SUI in female rats [55]. wBM-hMSC could be a valid alternative to the use of adipose BM-MSCs, especially in our expansion protocol which allows to collect larger amounts of cells to be transplanted. Since our study was limited to investigate the feasibility, the survival and the absence of side effects *in vivo*, further studies are needed to investigate the functional outcome, and will be performed in humans.

A major problem in stem cell therapy is the large amount of cells to be transplanted, whereas density gradient purification used in other studies causes a huge loss of cells and requires the collection of several mL of bone marrow under anesthesia. Our recent data demonstrate that it is possible to isolate and obtain a great expansion of hMSC from whole bone marrow, avoiding initial gradient separation, and with minimal *ex-vivo* manipulation [29]. Therefore, wBM hMSCs might be a very important, novel starting point in cell therapy for SUI treatment as patients, enrolled in a future clinical protocols, could be subjected only to a few ml BM aspirate, performed outpatient in local anesthesia, instead of surgical BM collection under total anesthesia.

In conclusion, some controversy still exists regarding the possibility of MSCs to differentiate into myoblasts *in situ* and fuse with the host myoblasts to form new myotubes. Our results clearly demonstrate that they can survive long and can migrate into the muscle, in absence of adverse side effects, i.e. they are not tumorigenic. Also, they do not need immunosuppressive therapy to survive. Therefore, MSCs are a promising tool for the treatment of SUI, since they are safe and not immunogenic, can provide a trophic and immunomodulatory support for the host. Further studies, performed in experimental models of disease, will elucidate the behavior and the role of MSC transplantation in a diseased environment.
